# Machine Learning Discovery of Record‐Low Lattice Thermal Conductivity in Double Perovskites

**DOI:** 10.1002/advs.202515766

**Published:** 2026-02-17

**Authors:** Md Zaibul Anam, Alejandro Rodriguez, Riccardo Rurali, Ming Hu

**Affiliations:** ^1^ Department of Mechanical Engineering University of South Carolina Columbia South Carolina USA; ^2^ Institut De Ciència de Materials de Barcelona ICMAB–CSIC Campus UAB Bellaterra Spain

**Keywords:** double perovskites, high‐throughput DFT, machine learning, phonon Boltzmann transport, record‐low lattice thermal conductivity

## Abstract

Double perovskites (ABC_2_D_6_) are versatile materials with applications in photovoltaics, optoelectronics, and thermoelectrics, where phonon‐mediated thermal transport is critical. However, high‐throughput phonon calculations by density functional theory (DFT) are computationally prohibitive due to the large supercells required. We develop a deep learning interatomic potential, Elemental‐SDNNFF, trained directly on DFT‐calculated forces within an active learning framework, enabling efficient prediction of phonon properties across thousands of double perovskites. Using this model, we screened 9709 cubic double perovskite structures, identifying 1597 dynamically stable candidates. Their lattice thermal conductivities (LTCs) were predicted by coupling Elemental‐SDNNFF with the Boltzmann Transport Equation, including off‐diagonal contributions. For the most promising compounds, DFT validation and four‐phonon scattering calculations revealed ultralow LTCs (<0.1 Wm^−1^K^−1^). Remarkably, Cs_2_HgPtCl_6_ was found to possess a bandgap of 0.35 eV and an LTC of 0.071 Wm^−1^K^−1^ at room temperature—the lowest ever reported for isotropic bulk materials, comparable to air. The result was independently confirmed by molecular dynamics simulations with a DeePMD potential and phonon lifetime extraction using DynaPhoPy. This work establishes an efficient machine learning‐assisted framework for fast screening of dynamic stability and accurate prediction of phonon transport in complex materials, highlighting double perovskites as Double Perovskites, High‐Throughput DFT, Machine Learning, Phonon Boltzmann Transport, Record‐Low Lattice Thermal Conductivity promising candidates for thermoelectric and thermal insulation applications.

## Graphical abstract

A deep learning interatomic potential is introduced to predict forces for computing phonon properties and thermal transport behavior in double perovskites. Screening 9,709 compounds identifies 1,597 stable materials, and Boltzmann transport calculations including both three and four‐phonon scattering suggests a record‐low lattice thermal conductivity of 0.071Wm‐1K‐1 for Cs_2_HgPtCl_6_, which is comparable to air.

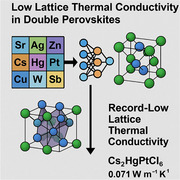

## 1 Introduction

The pursuit of novel materials with desired functionalities has become a significant field of study in recent years. Demands across different sectors such as energy, electronics, catalysis, and others have made material scientists look for suitable materials for a defined use case. Perovskites materials have gained significant attention in solar cells, light‐emitting diodes, photodetectors, and sensors fields [1, 2, 3, 4]. Single perovskites having generic formula of ABX_3_ are the prime candidates for these kinds of material properties [5, 6]. Despite having significant advantages, these perovskites pose a threat to the environment associated with lead‐based perovskites [7]. Additionally, single perovskites often suffer from stability issues, particularly under environmental stressors like moisture and heat, leading to degradation of their optoelectronic properties [8, 9]. Double perovskites are the best alternative to address these concerns [7]. Chen et al. showed that double perovskites have improved thermal stability and efficiency [10]. Double perovskites have a generic formula of ABC_2_D_6_, where A, B and C sites are cations, and D sites are anions. In double perovskite structures, A and B sites have a sixfold coordination, C site has a 12‐fold coordination [11]. The extraordinary tunability of double perovskites arises from the combinatorial possibilities of cation substitution at the A and B sites and their sensitivity to lattice distortions, octahedral tilting, and electronic interactions [12, 13]. These have made double perovskites a great candidate and a rich platform for applications in energy conversion and storage such as photovoltaics, optoelectronics, thermoelectrics, fuel cells, lithium batteries, and others [14, 15, 16].

For any material used in a practical application, material stabilities are always a concern. Lattice dynamic stability is one of them and refers to stability under small perturbations by vibrations of atoms within a crystal structure. A dynamically stable crystal's potential energy will increase with any small atomic displacement, and for a structure to be dynamically stable, all phonon frequencies throughout the Brillouin zone must be real and positive [17]. The absence of imaginary frequencies implies that the structure will not collapse, indefinitely departing from the static equilibrium point, under small perturbation of the atoms. Positive phonon dispersion serves as a predictive tool to design stable compounds, such as double perovskites, reducing the need for experimental testing. Investigation of properties of double perovskites demands dynamic stability check beforehand, as witnessed by several prior studies [18, 19, 20].

Moreover, to use double perovskites in energy conversion field such as thermoelectric applications [20, 21, 22, 23, 24], the dimensionless figure‐of‐merit, namely *ZT*  = *S* ^2^ σ*T*/ κ_*total*_ is usually used to evaluate the energy conversion performance of thermoelectrics, where *S* is the Seeback coefficient, σ is the electrical conductivity, κ_*total*_ is the total thermal conductivity which includes two components namely electronic (κ_*el*_) and phononic (κ_*p*_) thermal conductivity, and *T* is the absolute temperature. From this definition, it is evident that an excellent thermoelectric device should have high σ and *S*, but low κ [25]. There are many factors that can affect *ZT* coefficient such as carrier concentrations, band structure, phonon behavior, and others [26]. Among these parameters, the phononic (lattice) thermal conductivity is the only parameter that does not depend significantly on the electronic structure of a thermoelectric material. Many studies have showed that double perovskites possess ultralow lattice thermal conductivity (LTC), which provides the strong motivation to further pursue these materials in thermoelectric applications [27, 28, 29, 30, 31]. Considering large composition space of combinations for double perovskite formula ABC_2_D_6_, quickly predicting phononic (κ_*p*_) thermal conductivity of a large number of potential double perovskites is necessary.

In this study, a high‐throughput prediction of comprehensive phonon properties of 9709 double perovskite materials has been conducted to screen dynamically stable structures and ultralow thermal conductivity. Density functional theory (DFT) calculated atomic forces are utilized to train our recently developed elemental spatial density neural network force field dubbed as “Elemental‐SDNNFF” model. The newly trained Elemental‐SDNNFF model has demonstrated a competitive performance compared to the recently reported universal interatomic potentials [32]. In addition, based on the fact that this model previously has made a very competitive prediction for thermal conductivities [33], the phonon Boltzmann Transport Equation (BTE) approach was then employed to calculate the lattice thermal conductivities of the filtered 1597 dynamically stable double perovskites. Coherence phonon transport stemming from wave tunneling effect has also been included to get more accurate results of overall lattice thermal conductivity. Furthermore, four‐phonon scattering in the BTE calculation of selected materials has been conducted and the results show that adding higher order phonon scattering mechanism results in significant reduction in the overall thermal conductivity, leading to several extremely low thermal conductivity less than 0.1 Wm^−1^K^−1^. Afterwards, Cs_2_HgPtCl_6_ structure, which has a non‐zero bandgap, has been selected for further validation of the result. Deep Potential Molecular Dynamics (DeePMD) [34], a neural network potential, has showed DFT level accuracy in several studies [35, 36, 37]. Therefore, to evaluate the thermal conductivity results, at first, a DeePMD potential was trained using structural DFT data of Cs_2_HgPtCl_6_. This potential is then employed in Molecular Dynamics (MD) simulation to generate atomic trajectories, which subsequently analyzed using DynaPhoPy [38] to extract the material's total thermal conductivity. This result shows strong agreement with the results calculated using BTE, further validating the reliability of the results.

## 2 Results and Discussion

The high‐fidelity Elemental‐SDNNFF model is trained on two sets of datasets, namely initial pool and active learning pool. The double perovskites dataset includes 9709 cubic structures (material symmetry $Fm\bar{3}m$ and space group no. 225) covering 61 elements in total across periodic table, with detailed distribution of number of structures composed of different elements shown in Figure S1, and detailed statistics of A, B, C, and D sites is provided in Figure S2. In a typical ABC_2_D_6_ type double perovskites, A and B are small cations, and C is larger compared to A and B [39]. According to the dataset statistics shown in Figure S2a, the A and B site is mostly occupied by small alkali materials like Li and Na, along with many transitional materials Sc, Cu, Ni and other elements. In contrast, C site elements reside in the center of the cubooctahedral cage [40], and it is largely filled by large monovalent and divalent cations such as Cs, Rb, K, and Ba, as illustrated in Figure S2b. This diverse set of elements at C site gives the freedom to choose A and B site cations with oxidation states ranging from one to seven, contributing to the wide chemical diversity of A and B site elements across the periodic table [39]. As shown in Figure S2c, the D site is normally dominated by halides and other nonmetallic elements such as O, H, S, and others [41, 42, 43]. This distribution reflects that most double perovskites used in this study are composed of halides and chalcogenides. These are ideal ionic species to form octahedra where voids are filled by C site cations. This octahedra can expand or contract depending on the size and charge of neighboring A and B site cations [39]. After collecting an initial pool of 9709 double perovskites in cubic phases, 16 396 displaced atomic configurations from their optimized position with their corresponding forces are used as initial training dataset, which was accumulated in our recent studies. [44, 45, 46, 47, 48] Another 4037 displaced atomic configuration from their optimized position with their corresponding forces are incorporated in five active learning rounds, resulting in a total of 20 433 atomic configurations used in training for the finalized deep learning model for production run. The details about dataset creation and active learning process are given in the methodology section. After one initial training round, and five active learning rounds, making in total of six training rounds, the Elemental‐SDNNFF model is improved by incorporating new data from the most uncertain atomic configurations in each round.

**FIGURE 1:**
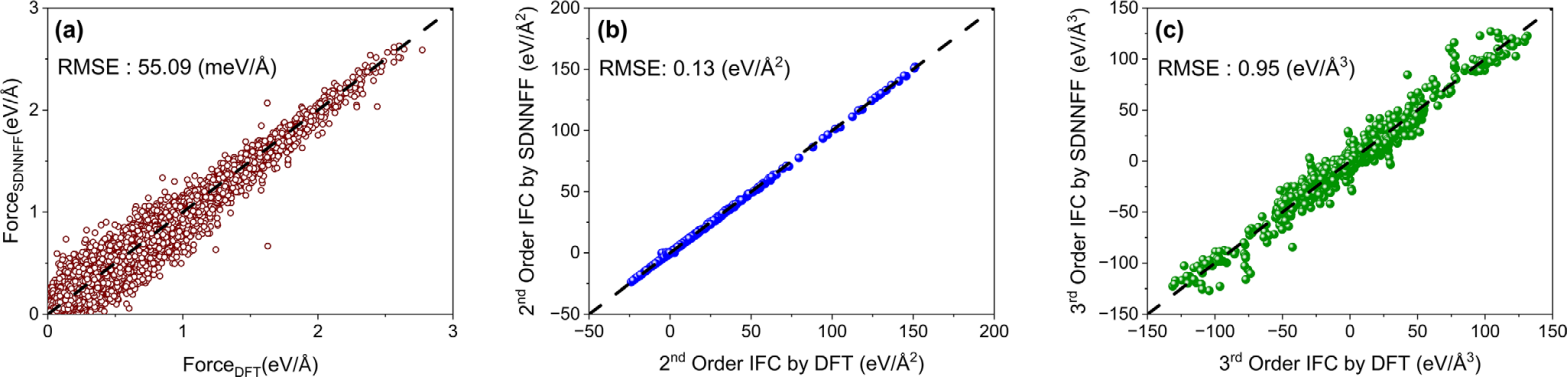
(a) Comparison of atomic force predictions by Elemental‐SDNNFF model and DFT values. Each red dot represents a single force component. (b) Second and (c) third order interatomic force constants (IFCs) fitted using these forces compared to DFT calculations for 118 selected structures. The dashed lines represent the ideal match with DFT values and are guide for eyes.

After the sixth round of deep learning training, 6157 displaced atomic configurations, which are not seen in initial training and any active learning training rounds, are used to evaluate atomic forces using the finally frozen Elemental‐SDNNFF model. These new configurations are chosen as they had the most uncertainty after the fifth round of training and therefore, they are a good choice for evaluating the performance of the finalized model. Figure 1a show the comparison of evaluated atomic forces of these 6157 atomic configurations by our Elemental‐SDNNFF model and DFT calculations. It is evident that the Elemental‐SDNNFF model has excellent performance in terms of the root mean squared error (RMSE) of predicted forces of 55.1 meV/Å, and a high coefficient of determination (R^2^) value of 0.98. Such low RMSE and high R^2^ value in predicted atomic forces is competitive considering that our Elemental‐SDNNFF model covers 61 diverse elements across the periodic table, unlike the previous MLPs that were only trained on a single material or material family [49, 50, 51, 52].

We also considered and compared with some recent developments in universal machine learning potentials (uMLPs). Herewith, we selected two widely used ones, namely pretrained CHGNet v0.3.0 model for CHGNet [53] and MACE‐MPA‐0 model for MACE [54], and evaluated the forces for the same dataset of 6157 atomic configurations. The same comparisons are then made and shown in Figure S3. It is evident from the comparison that Elemental‐SDNNFF has a much lower RMSE compared to the other two uMLPs. The predictive performance of machine learning models highly depends on the type and accuracy of the training dataset [55]. CHGNet and MACE pretrained models are trained on a diverse Materials Project trajectory [56] (MPTrj) datasets, which makes them versatile across a wide range of materials, but less optimized for a specific material family like double perovskites herein. On the other hand, Elemental‐SDNNFF model is exclusively trained on the double perovskites structures, which are taken from Open Quantum Materials Database (OQMD) [57]. Also, the performance of the Elemental‐SDNNFF model was improved through active‐learning, as detailed in the methodology section. Another important factor is the large supercells (total number of atoms in the range of 80–300) used here for Elemental‐SDNNFF model, which pairs local atomic environments to the central atom for forces, creating *N* (total number of atoms in the cell) datapoint for a single supercell. Consequently, uMLPs rely on singular energy value per supercell as their training data and may introduce forces and virial stresses in the loss function. This treatment invokes a singular update of MLP parameters per supercell typically by a summation of all atomic forces and virial terms. This contrasts with Elemental‐SDNNFF model where the model's parameters are updated with each central atom force, and no important atomic force information is lost due to summation of forces seen in the uMLPs, providing a significant advantage in force prediction required for phonon properties. This perspective is supported by Focassio et al., who showed that specialized MLPs demonstrate high accuracy predictions compared to CHGNet and MACE models [58].

Following the evaluation of the forces for all 9709 double perovskite structures, excluding all the atomic configurations used in model training, the interatomic force constants (IFCs) are fitted up to third order using the predicted forces [59]. Ordinary Least Square (OLS) fitting technique is used to fit the IFCs. After IFC fitting, dynamic stability of the structures is checked, and 1597 out of the total 9709 structures are found to be dynamically stable, that is, there is no imaginary frequency found in the first Brillouin zone (BZ) with frequency threshold of −0.01 THz. The quality of the fitted IFCs using OLS technique is highly reliable to the predicted forces [60]. To check the quality of IFC fitting using the predicted forces by our Elemental‐SDNNFF model, 118 predicted dynamically stable double perovskite structures are randomly selected out of the total 1597 structures. IFC fitting is done for these structures with DFT calculated forces and then compared with IFCs fitted using forces predicted by our Elemental‐SDNNFF model. The second order IFC is basically composed of 3 × 3 matrix for each atom pair, and the third order IFC is composed of 3 × 3 × 3 tensor representing each triplet of interacting atoms. For second order IFC comparison, the trace of the matrix for each pair of atoms is taken. For third order IFCs, specific components corresponding to diagonal elements, such as (1,1,1), (2,2,2) are selected from 3 × 3 × 3 tensor for each triplet. In Figure 1b,c, comparisons for the second and third order IFCs between our Elemental‐SDNNFF model and full DFT calculation are shown. The second order IFCs exhibit excellent agreement with DFT with an RMSE as low as 0.13 eV/Å^2^, reflecting the model's capability to accurately capture harmonic interactions. Figure 1c shows that the RMSE for third order IFCs is slightly increased to 0.95 eV/Å^3^ compared to second order IFCs, which shows the complexity and sensitivity involved in predicting anharmonic interactions. These results are highly competitive, with the accuracy of the IFCs exceeding that of the uMLPs, as shown in Figure S3e–f,h,i. Nevertheless, this accuracy shows strong predictive performance of Elemental‐SDNNFF model for anharmonic IFCs for the 118 selected structures.

Once dynamically stable materials have been screened and their IFCs are obtained, the IFCs are used to calculate the lattice thermal conductivity. Phonon BTE coupled with DFT calculations is one of the best approaches to study particle‐like phonon transport called propagons [61, 62, 63, 64]. By solving BTE, the phononic or lattice thermal conductivity (κ_*p*_) can be written as [65].

(1) $$\kappa_{p}^{\alpha \beta }=\frac{1}{NV}\sum_{\omega }\frac{\partial n_{\omega }}{\partial T}\left(\hbar \omega \right)v_{\omega }^{\alpha }v_{\omega }^{\beta }\tau_{\omega }$$

where $n_{\omega }$ is the phonon mode with frequency ω dependent occupation probability, *N* is total number of phonon modes, *T* is absolute temperature, τ_ω_ is relaxation time, *v*_ω_ is phonon group velocity, and α, β is cartesian component direction (*x*, *y*, or *z*). Scattering rate τ_ω_ is the most important factor in determining κ_*p*_ and it can be written as [66].

(2) $$\frac{1}{\tau_{\omega }}=\frac{1}{N}\;\left[\sum_{\omega^{\prime }\omega^{\prime \prime }}^{+}\Gamma_{\omega \omega^{\prime }\omega^{\prime \prime }}^{+}+\sum_{\omega^{\prime }\omega^{\prime \prime }}^{-}\Gamma_{\omega \omega^{\prime }\omega^{\prime \prime }}^{-}\right]$$

where Γ^+^, Γ^−^ represents the phonon absorption and emission process in three‐phonon scattering, respectively. ShengBTE [66] package is used in this study to calculate the LTC of the double perovskite structures.

**FIGURE 2:**
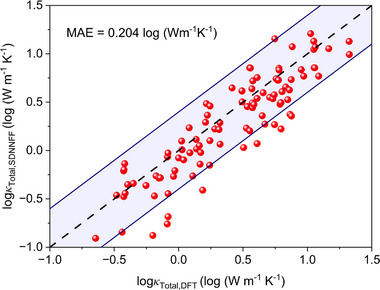
Comparison of total thermal conductivity (κ_*total*_, including both propagons and diffusons) between DFT and Elemental‐SNDNNFF model prediction on 100 dynamically stable structures. The dashed line represents perfect agreement with DFT values, while the shaded region illustrates the 90% confidence interval.

In recent years, several studies have showed the importance of contribution from non‐diagonal terms of heat flux operators (called diffusions) for ultralow LTC materials. [67, 68, 69, 70, 71] For double perovskites whose intrinsic LTC might be very low, the off‐diagonal contribution to overall thermal transport is crucial due to their complex crystal structures, which could lead to strong anharmonicity and coupling between phonons in different directions. The total thermal conductivity can then be written as, κ_*total*_ = κ_*p*_  + κ_*c*_, where the off‐diagonal contribution or coherence part (κ_*c*_) can be calculated as, [68]

(3)

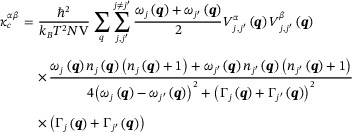

where *k_B_* is Boltzmann constant, *V* is the volume of the unit cell, *T* is the absolute temperature. In this study, both κ_*p*_ and κ_*c*_ are calculated at 300 K for all 1597 dynamically stable double perovskite structures, then κ_*total*_ is obtained for all these structures. The logarithm value of κ_*total*_ for 100 selected double perovskites structures by direct DFT calculation and our Elemental‐SDNNFF model prediction are compared in Figure 2. The comparison shows a mean absolute error (MAE) of 0.204 log (Wm^−1^K^−1^), which is comparable to the previous work [33]. It is worth noting that, this MAE is competitive with the results achieved in other works. [72, 73, 74] Figure 2 indicates that the model captures higher κ_*total*_ values more accurately than the lower ones. Usually, lower κ_*total*_ values are due to higher phonon scattering, which presents strong anharmonicity. [75] These anharmonic effects are generally characterized using third order IFCs. As the accuracy of the predicted third order IFCs is lower compared to second order IFCs, as shown in Figure 1, which contributes to increased errors in the prediction of low LTC regions.

**FIGURE 3:**
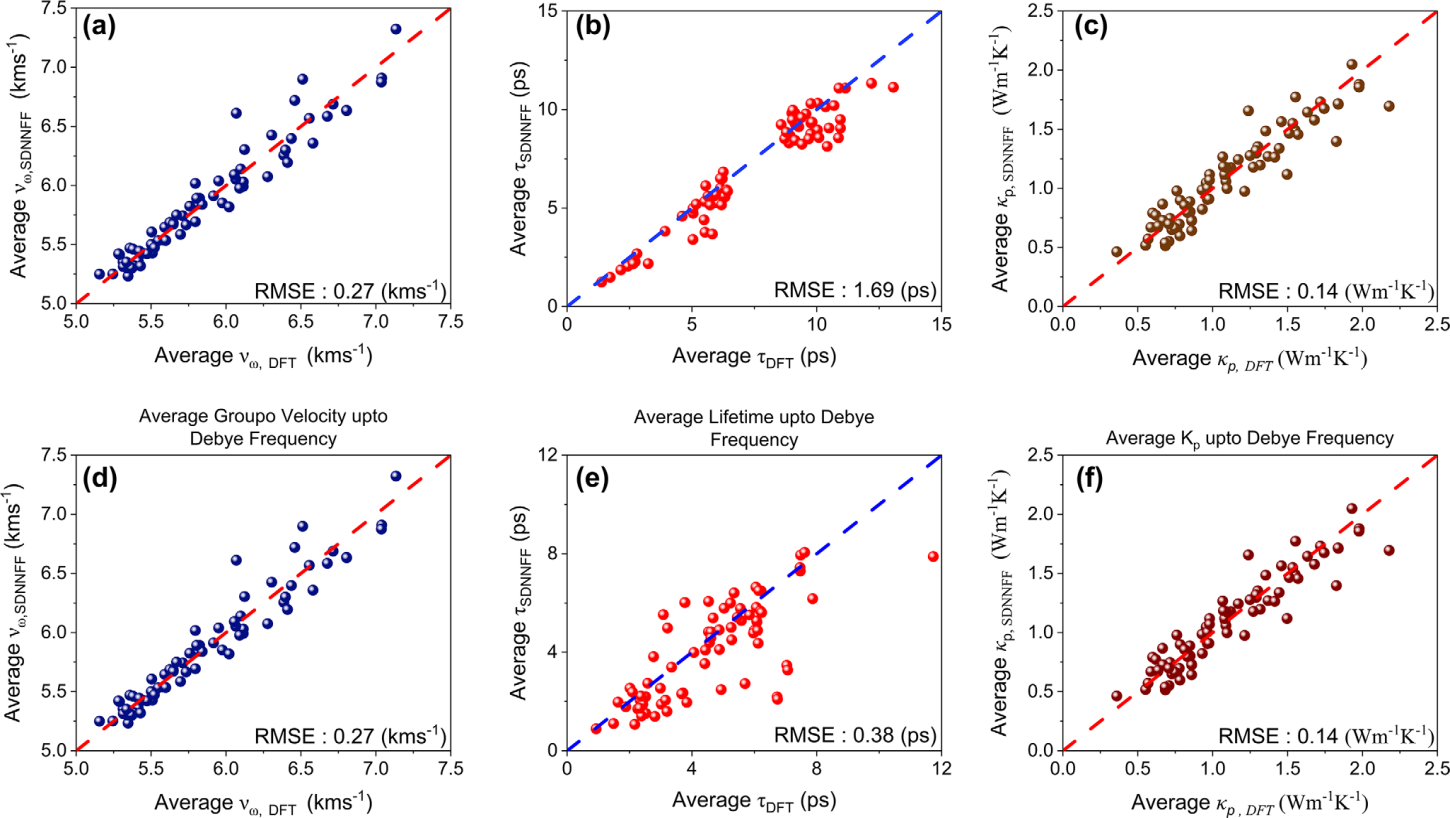
Comparison between Elemental‐SDNNFF and DFT calculated (a), (d) average phonon group velocity (*v*_ω_), (b), (e) average phonon lifetime (τ), and (c), (f) average thermal conductivity (propagons, κ_*p*_). The results for the top and bottom row are calculated for all phonon modes and up to Debye frequency, respectively. See the texts for details.

From Equation (1), it is evident that the phononic or lattice thermal conductivity (κ_*p*_) is governed by group velocity and lifetime of the phonons. [76, 77] Thus, the accuracy of these two properties may significantly influence the results of κ_*p*_. To this end, in Figure 3a,b we show the comparison between DFT and Elemental‐SDNNFF predicted average group velocity (*v*_ω_) and average phonon lifetime (τ), respectively. Since both properties are phonon mode dependent, in case of Figure 3a,b, all phonon modes are considered for a single structure to calculate the average *v*_ω_ and τ. A similar comparison is shown in Figure 3d,e, however, in this case, only the phonon modes up to the Debye frequency of the structure are considered for obtaining the average *v*_ω_ and τ. By comparing the two results, it is evident that the RMSE for average group velocity remains the same for both cases in Figure 3a–d. However, the RMSE for average phonon lifetime changes significantly for the cases in Figure 3b–e. Generally speaking, optical phonons have low group velocity due to the flat band features in phonon dispersions and thus contribute less to overall thermal conductivity. [78, 79] Therefore, phonon modes with frequency greater than Debye frequency have little contribution to the average calculation for phonon group velocity, resulting in almost similar RMSE result for both cases shown in Figure 3a–d. This is also valid for mode dependent thermal conductivity calculation shown in Figure 3c–f, where the RMSE for both cases are almost similar. In contrast to these results, the phonon lifetime is highly dependent on phonon anharmonicity and phonon band gaps in the band structure. [77] Phonon–phonon interactions and large band gaps can significantly change the lifetime of optical phonons, and this is why in Figure 3b–e the difference in RMSE is apparent. The results from Figure 3b–e also imply that phonon lifetime for optical phonons are generally not negligibly small, which can be seen from the overall upshift in the magnitude of average values by comparing Figure 3e,b, suggesting strong phonon anharmonicity of the optical phonons in double perovskites. However, those high frequency optical phonon modes do not contribute much to overall thermal transport due to their limited group velocity, according to Equation (1).

**FIGURE 4:**
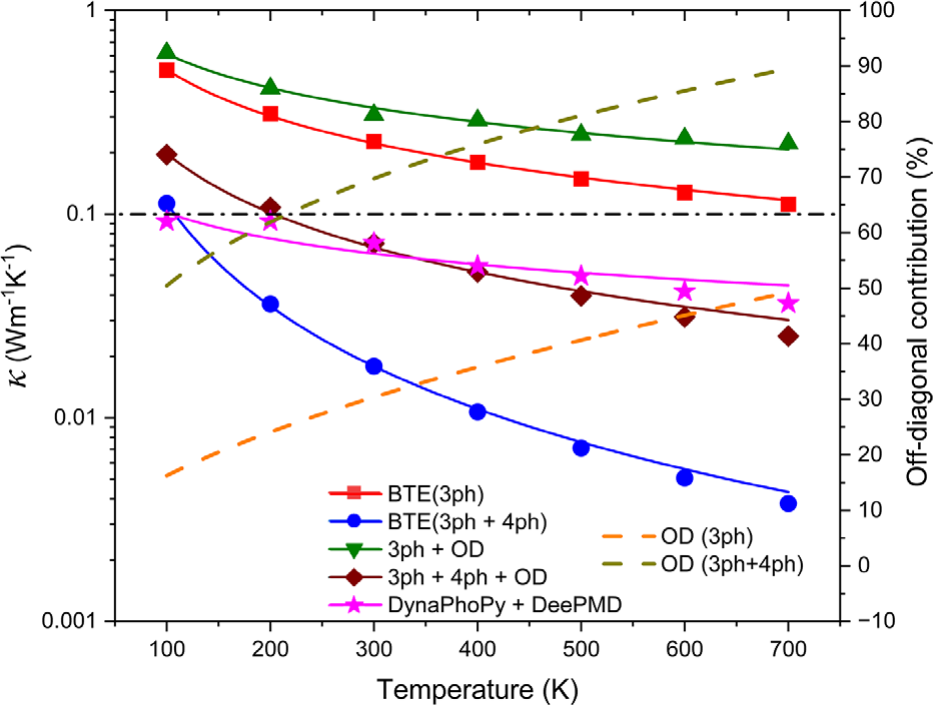
(Left axis) Temperature dependent total thermal conductivity (κ_*total*_ = κ_*p*_  + κ_*c*_) of Cs_2_HgPtCl_6_ computed under different phonon scattering conditions. Red and blue curves represent only propagons (κ_*p*_) values, whereas green and brown curves include the off‐diagonal (OD) thermal conductivity contributions (κ_*c*_). Four‐phonon (4 ph) scattering along with three‐phonon (3 ph) condition is considered only for blue and brown curves, and the other two curves represent the 3 ph process only. The dashed lines indicate the off‐diagonal contribution percentage (right axis). The horizontal dashed‐dot line represents total thermal conductivity of 0.1 Wm^−1^K^−1^. The magenta curve represents the total thermal conductivity results obtained by MLP‐equipped MD simulations with phonon relaxation time extracted from atomic trajectories (see text for details).

After getting κ_*total*_ for all dynamically stable OQMD structures which are derived from the predicted forces of Elemental‐SDNNFF model, a subset of 47 structures with ultralow thermal conductivity (κ_*total*_ < 0.1 Wm^−1^K^−1^, including both propagons and diffusions) are selected to run additional DFT calculations for validation. The DFT calculated results indicate that none of the structures exhibit a κ_*total*_ below 0.1 Wm^−1^K^−1^. In all these calculations, we have only considered three‐phonon (3 ph) process for phonon–phonon scattering mechanism while calculating κ_*total*_ with ShengBTE. However, Feng et al. has shown that calculations with 3 ph scattering can sometimes overestimate LTC values, and introducing four‐phonon (4 ph) scattering in the calculation can significantly decrease the LTC values [80]. This observation has been supported by several other studies, [69, 81, 82, 83] showing the importance of incorporating 4 ph interactions in thermal transport calculations. Considering this reduction in LTC value, we included 4 ph scattering in addition to 3 ph for BTE calculation in ShengBTE for all 47 structures. We further calculated the corresponding off‐diagonal contribution after running 4‐phonon BTE. The full LTC information for all 47 structures before and after considering 4‐phonon scattering are shown in Table S1. We finally found that Cs_2_HgPtCl_6_ possesses the lowest *κ_t__o__t__a__l_* among all 47 structures after considering 4‐phonon scattering. To perform more comprehensive comparative analysis, five representative structures were then selected, including the two lowest LTC structures, as shown in Figure S4. For this, at first, IFCs up to fourth order are generated using OLS method using the similar technique discussed above. In this case, we used the extension of original ShengBTE package developed by Feng et al. [84] which includes the 4 ph scattering rate in thermal conductivity calculations. Figure S4 illustrates the comparison of different thermal conductivities for these selected five structures based on different scattering considerations. Figure S4b indicates that for all structures, inclusion of 4 ph process significantly reduces the LTC (κ_*p*_) values compared to only 3 ph process, particularly for Cs_2_HgPtCl_6_ and Cs_2_KRhCl_6_. However, as illustrated in Figure S4a, κ_*total*_ values for structures Cs_2_KIrCl_6_, Cs_2_KRhCl_6_, and Cs_2_InRhBr_6_ have increased upon inclusion of the 4 ph scattering process. This contradictory behavior is attributed to the inclusion of off‐diagonal or diffusions contribution (κ_*c*_) in the κ_*total*_ values. As shown in Figure S4c, κ_*c*_ values have increased significantly when 4 ph process is considered for these three structures, compensating for the drop in κ_*p*_ and resulting in overall increase in κ_*total*_ values. On the other hand, Figure 4a indicates that, for Cs_2_HgPtCl_6_ and Cs_2_AgOsBr_6_, the κ_*total*_ values have dropped below 0.1 Wm^−1^K^−1^ (0.071 and 0.063 Wm^−1^K^−1^, respectively). We continue to calculate the electronic band structure of these two materials and only Cs_2_HgPtCl_6_ exhibits considerable bandgap of 0.35 eV, consistent with the OQMD reported value of 0.418 eV. To the best of our knowledge, no experimental reports on this material have been published. Therefore, we only consider Cs_2_HgPtCl_6_ for further analysis and validation.

As shown in Figure 4, it is evident that the inclusion of the 4 ph process on top of 3 ph process leads to a noticeable reduction in total thermal conductivity for Cs_2_HgPtCl_6_ at a wide range of temperatures (100–700 K studied herein). Even after considering the off‐diagonal contribution (κ_*c*_) to the total thermal conductivity, approximately after 300 K the κ_*total*_ values are lower than 0.1 Wm^−1^K^−1^. Specifically, both κ_*p*_ and κ_*c*_ of Cs_2_HgPtCl_6_ reaches unprecedently low value of 0.018 and 0.053 Wm^−1^K^−1^ at room temperature, respectively, yielding κ_*total*_ of 0.071 Wm^−1^K^−1^. This record‐low LTC for a pristine isotropic bulk inorganic crystal is just 3 times the thermal conductivity of air. The percentage of the off‐diagonal contribution for both 3 ph and 3ph + 4 ph scattering case is shown by dashed lines in Figure 4. As Cs_2_HgPtCl_6_ is an ultralow thermal conductivity material, the wave‐like properties are dominant, causing high percentage of off‐diagonal contribution to total thermal conductivity. The off‐diagonal contribution for 3ph + 4 ph scattering is much higher compared to only 3 ph case, e.g., approximately 70% versus 30% at 300 K, respectively. A similar trend is also found in thermal transport in Cs_2_AgBiBr_6_ by Zheng et al. [69]

**FIGURE 5:**
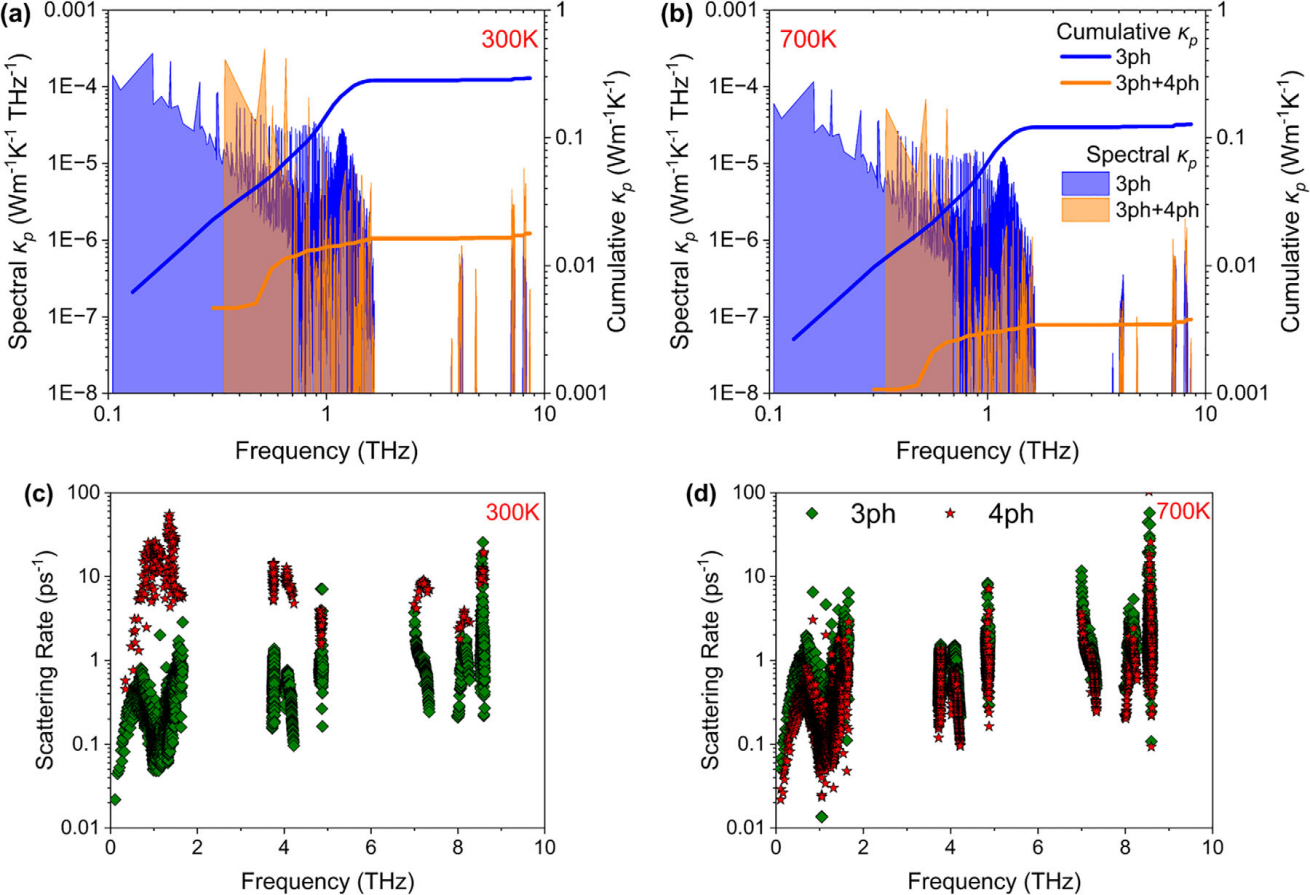
Mode dependent spectral (left axis) and cumulative lattice thermal conductivity (right axis) at (a) 300 K and (b) 700 K for 3 ph and 3ph+4 ph interactions. All axes are presented on a logarithmic scale for better visualization. Similarly, mode dependent scattering rates at (c) 300 K and (d) 700 K for separate 3 and 4 ph interactions.

For Cs_2_HgPtCl_6_, propagons contribution (κ_*p*_) to overall thermal conductivity is mostly carried by phonons with frequency lower than 2 THz for both three and four phonon cases, as observed from Figure 5a,b. In case of higher temperature (700 K) and four phonon shown in Figure 5b, the phonon population that mostly contribute to LTC are found to be below 1 THz. This lower frequency region is mainly attributed to acoustic phonons, and some of the low energy optical phonons, which is analogous to the previous studies [69, 85]. Some higher frequency range (7–8 THz) optical phonons also contribute to particle‐like phonon propagation in case of four phonons which can be observed from Figure 5a,b, however, this population is not significant. For both temperatures shown in Figure 5a,b, the cumulative LTC dropped significantly in four phonon cases. This is due to the high scattering rate integrated when considering four phonon interaction, which is depicted in Figure 5c,d. The significant reduction of κ_*p*_ by considering 4‐phonon scattering implies that there is very strong higher order phonon anharmonicity in Cs_2_HgPtCl_6_, in particular for phonon modes with frequency less than 2 THz.

**FIGURE 6:**
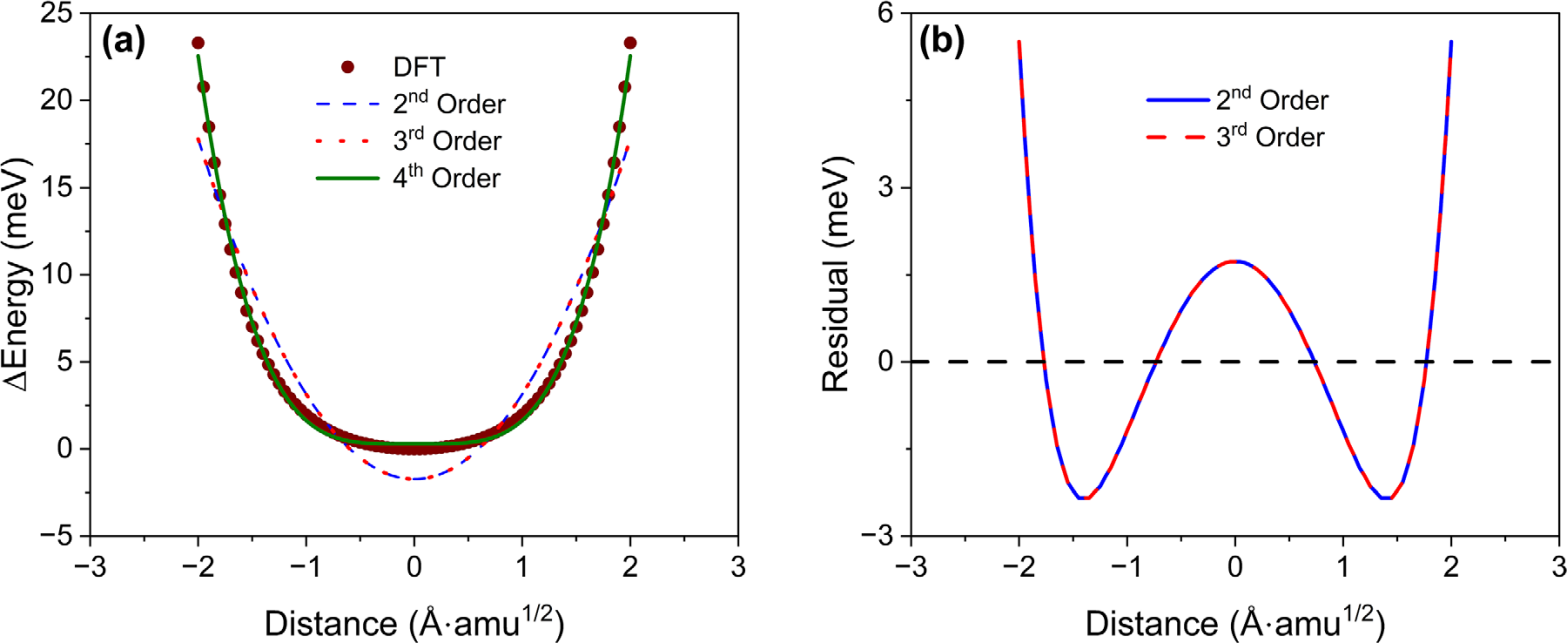
(a) DFT calculated potential energy surface for a representative optical phonon mode at Γ‐point with corresponding frequency of ∼1.5 THz. The original potential energy of the displaced primitive cell calculated by DFT is represented by solid dark red symbols. The blue dashed line, red dot line, and green solid lines represent second, third, and fourth order polynomial fitting of the potential energy landscape, respectively. The distance (Å·amu^1/2^) in the plot represents the normal mode coordinates. (b) The residual curve of the potential energy by subtracting the second and third order polynomial fitting from the original potential energy.

To provide direct evidence of strong phonon anharmonicity of Cs_2_HgPtCl_6_, first, additional DFT calculations of the local potential energy landscape are performed for a representative optical phonon mode at Γ point with corresponding frequency of ∼1.5 THz. The potential energy surface is evaluated over a wide range of normal mode coordinates constructed from the eigenvectors of the selected phonon mode. Figure 6a illustrates that quadratic (second order) and cubic (third order) polynomial fitting cannot capture the energy landscape properly, while quartic (fourth order) polynomial provides an excellent fit to the DFT results. This quartic anharmonicity behavior is further confirmed through the residual analysis technique [86], where the quadratic and cubic polynomials fits are subtracted from the original potential energy curve, as shown in Figure 6b. The resulting “W”‐shaped residual curve indicates the presence of fourth order phonon anharmonicity in Cs_2_HgPtCl_6_. Second, to further understand the underlying mechanism from electronic level analysis, crystal orbital Hamilton population (COHP) and integrated COHP (ICOHP) are utilized to explain the phonon transport behavior for Cs_2_HgPtCl_6_ as both are good indicators for bonding strength and anharmonicity, which affect LTC [87, 88, 89, 90, 91, 92]. Antibonding COHP states under the Fermi energy (ε_F_) proved to be a good indicator of strong anharmonicity and low LTCs in several studies [87, 88, 89, 90, 91], and from Figure S5 it is evident that COHP of Cs_2_HgPtCl_6_ has antibonding states under ε_F_ which indicates that the material has strong anharmonicity that could induce ultralow LTC. In addition, it has been reported that low values of normalized −ICOHP and normalized integrated crystal orbital bond index (ICOBI) are associated with low LTC and high anharmonicity [92]. The normalized −ICOHP reflects bond strength and lower values indicate weaker bonding, which generally leads to stronger anharmonic lattice vibrations. The normalized ICOBI represents the degree of covalent bonding in a material. Because covalent bonds are typically stronger than ionic or metallic bonds, a low normalized ICOBI value usually suggests weak covalent bonding and therefore enhanced anharmonicity [93]. For Cs_2_HgPtCl_6_, we calculated normalized −ICOHP and normalized ICOBI values of 0.779 eV and 0.101, respectively. These values are comparable to those reported for other low LTC materials by Al‐Fahdi et al. [92]. This confirms the presence of strong phonon anharmonicity and weak bonding in Cs_2_HgPtCl_6_, providing a clear explanation for its extremely low LTC.

**FIGURE 7:**
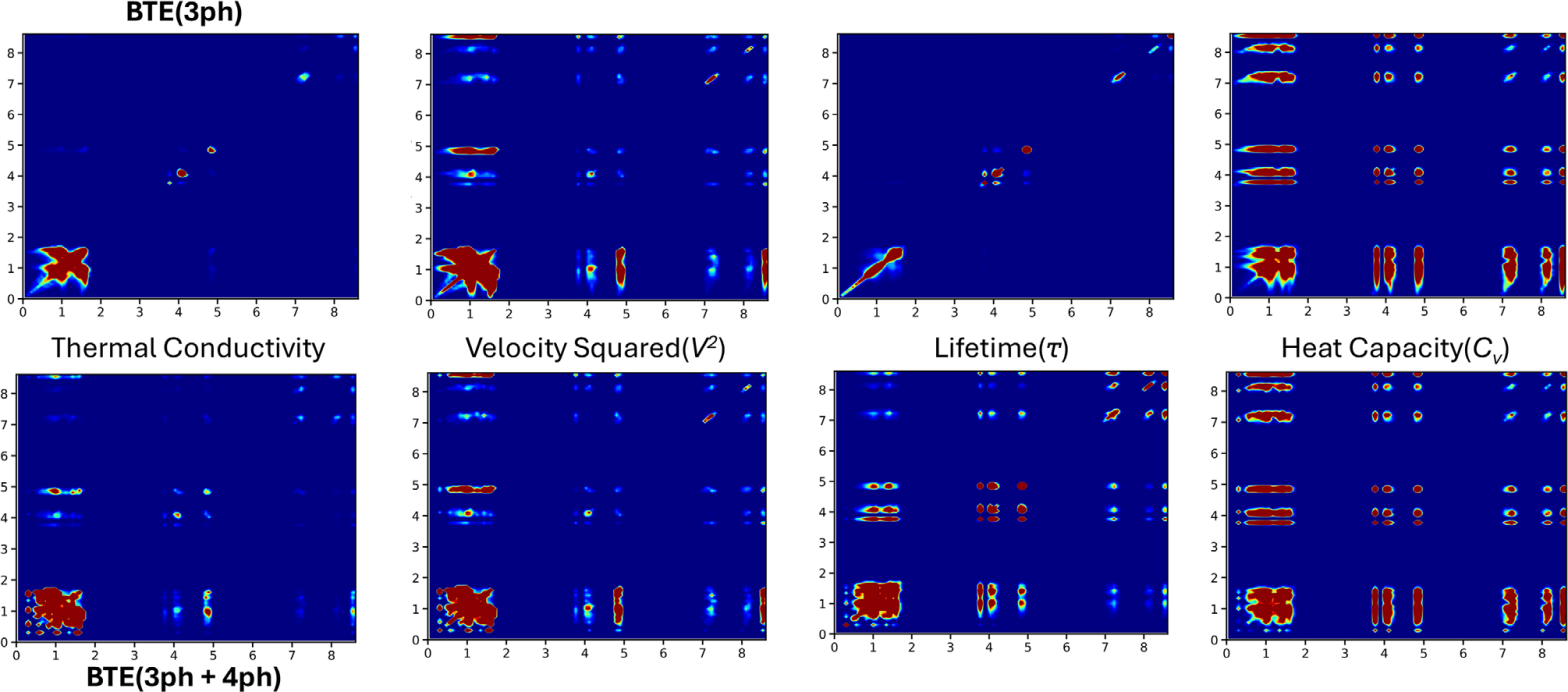
Mode dependent phonon property matrices calculated by only 3 ph process (top row) and 3ph + 4 ph process (bottom row) for Cs_2_HgPtCl_6_.

To further analyze the off‐diagonal contribution for Cs_2_HgPtCl_6_, a frequency dependent phonon property matrices such as thermal conductivity, velocity squared, lifetime, and heat capacity is shown in Figure 7. These matrices are generated by mapping the phonon properties to their corresponding *q*‐points along with their specific frequencies. This mapping allows us to project the values for these properties on a specific grid, and heat mapping the whole grid system shows where the values are dominant. By doing this, vivid representations of both diagonal and off‐diagonal parts can be observed directly in Figure 7. This illustrates that, when only three‐phonon interactions are considered, total thermal conductivity is mostly contributed by its diagonal values. The heat map shows that the values are mostly concentrated in the low frequency region, and very timid sparse diagonal values can be observed along the diagonal path. For this reason, these structures present a low thermal conductivity value, which has a few off‐diagonal contributions. Only diagonal values for this case can be attributed to lifetime, which is the most dominant property, and from its heat map, we can see that its value is mostly concentrated on the diagonal region. On the other hand, when considering both three and four phonon interactions, in Figure 7, it illustrates that the thermal conductivity values are scattered, meaning the overall thermal transport has more off‐diagonal contributions compared to only three phonon case. In this case, all the properties show an off‐diagonal values present, and thus it reflects in the thermal conductivity heat map. However, both diagonal and off‐diagonal contribution occurs in small regions, and the total matrix shows a very weak contribution, and this leads to ultralow thermal conductivity in this case.

**FIGURE 8:**
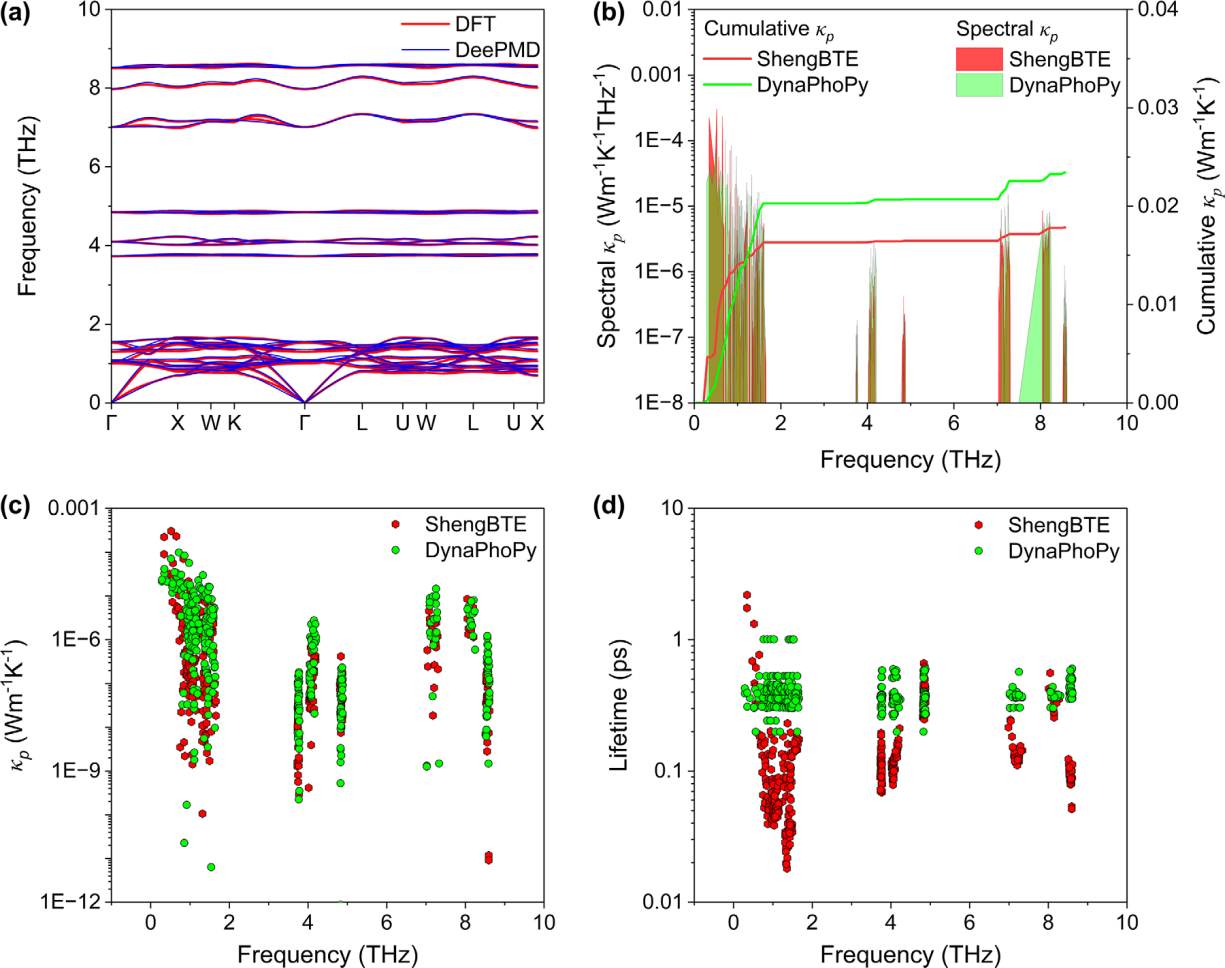
(a) Comparison of phonon dispersion curve using second order IFCs derived from DFT calculations and DeePMD potential. Comparison of thermal transport properties of Cs_2_HgPtCl_6_ at 300 K between computation of ShengBTE and DynaPhoPy in terms of (b) spectral (left axis) and cumulative (right axis) LTC as a function of frequency, (c) mode dependent LTC values, and (d) mode dependent phonon lifetime. For ShengBTE results, the lifetime shown considers 3ph + 4 ph scattering.

To further validate the unprecedentedly ultralow total thermal conductivity of Cs_2_HgPtCl_6_, we performed additional MD simulations with specifically trained DeePMD potential on structural data from Cs_2_HgPtCl_6_ and then DynaPhoPy package is used to analyze the MD trajectory data to determine phonon linewidths, from which phonon lifetime are subsequently extracted [94]. To ensure the consistency of the results with ShengBTE calculations, DynaPhoPy requires a supercell whose dimensions must match the selected *q*‐grid. Also, it requires the second order IFC fitted using the same supercell size. To this end, we used a large 6 × 6 × 6 supercell, totaling 2160 atoms for MD simulations and DeePMD potential is used to generate the second order IFC using OLS method. To verify the accuracy of this IFC, the phonon dispersion curves are generated using this IFC and directly compared with those obtained from the DFT‐fitted IFCs, as shown in Figure 8a. The phonon dispersion curve shows a great consistency of the two IFCs. The trend in Figure 8d indicates that the phonon lifetimes calculated using DynaPhoPy mostly fall within the range of 0.1–1 ps and show good agreement with the values obtained from ShengBTE calculations. Subsequently, phonon group velocities and heat capacity matrices are computed using harmonic approximation implemented in ShengBTE. These data allowed the calculation of both diagonal and off‐diagonal LTC values to finally calculate the total thermal conductivity of this material. The spectral LTC distribution in Figure 8b shows generally consistent frequency dependent behavior from ShengBTE and DynaPhoPy, mainly in the lower frequency regions, although the cumulative LTC curves by DynaPhoPy yields slightly higher κ_*p*_ compared to ShengBTE. Figure 8c further supports this observation where the mode dependent κ_*p*_ values are clustered in the same frequency range for both cases, with main contribution to overall phonon transport coming from low frequency phonon modes (less than 2 THz). Since this is an ultralow thermal conductive material, the off‐diagonal contribution is significant for this material as shown in Figure 7. So, after getting the phonon lifetime matrix from DynaPhoPy, the off‐diagonal values are calculated in a similar way as before, and for this case we found that κ_*c*_ ∼0.5 Wm^−1^K^−1^at 300 K. This κ_*c*_ attributes to almost 70% of total thermal conductivity at 300 K which is comparable to four phonon results obtained from ShengBTE. Finally, the overall thermal conductivity (κ_*total*_) of Cs_2_HgPtCl_6_ at 300 K including the off‐diagonal contributions, calculated using phonon lifetime from DynaPhoPy is 0.072 Wm^−1^K^−1^, which matches perfectly the value obtained from DFT+BTE approach (0.071 Wm^−1^K^−1^), validating the extremely low thermal conductivity found for the material. We continued to conduct MD simulations with the trained DeePMD potential for temperature ranging from 100 to 700 K and then use DynaPhoPy to extract the phonon lifetime and subsequently obtain the temperature dependent κ_*total*_ with the same procedure as above. The result is included in Figure 4 for comparison. The temperature dependent κ_*total*_ by DynaPhoPy+DeePMD matches well the values by DFT+BTE with full consideration of 3 ph, 4 ph, and off‐diagonal contributions. The MD simulations confirm Cs_2_HgPtCl_6_ indeed possesses extremely low lattice thermal conductivity over a wide temperature range. It is striking to see that the κ_*total*_ of Cs_2_HgPtCl_6_ at 700 K is as low as 0.035 Wm^−1^K^−1^, which is even lower than that of air at the same temperature (roughly 0.05 Wm^−1^K^−1^).

## 3 Conclusion

In summary, we developed a deep learning interatomic potential, Elemental‐SDNNFF, to enable high‐throughput screening of phonon transport properties in cubic double perovskites. By training directly on DFT‐calculated forces within an active learning framework, the model achieved high accuracy across 61 chemical elements and was applied to 9709 candidate structures, from which 1597 dynamically stable compounds were identified. Their lattice thermal conductivities were then predicted using the phonon Boltzmann Transport Equation with both diagonal (propagon) and off‐diagonal (coherence) contributions. Further validation with DFT calculations and inclusion of four‐phonon scattering revealed several compounds with ultralow LTCs (< 0.1 W m^−1^ K^−1^). Notably, Cs_2_HgPtCl_6_ was found to possess a finite bandgap of 0.35 eV and a record‐low LTC of 0.071 W m^−1^ K^−1^ at room temperature, comparable to that of air. The ultralow LTC of the double perovskite Cs_2_HgPtCl_6_ is mainly attributed to three factors: 1) extremely low group velocities induced by weak chemical bonding; 2) ultrashort phonon relaxation time induced by strong phonon anharmonicity in particular higher (fourth) order phonon anharmonicity for phonon modes with frequency less than 2 THz; and 3) both diagonal and off‐diagonal contribution occurring in small regions, leading to a very weak contribution of the total matrix to overall thermal transport. This unprecedented result was further independently confirmed by molecular dynamics simulations with deep learning interatomic potential (DeePMD) and phonon lifetime analysis using DynaPhoPy. Overall, this study establishes an efficient and generalizable machine learning‐assisted framework for quickly screening and predicting phonon transport in complex materials. It is also worth pointing out that the effectiveness of the current framework is closely related to the accuracy of the prediction by Elemental‐SDNNFF model, and Rodriguez et al. [33] have shown that it can reliably capture a diverse range of crystal chemistry. Beyond discovering a record‐setting thermal insulator, our findings highlight the promise of double perovskites as candidates for thermoelectric and thermal insulation applications, and this framework can be readily extended to other crystal families like single perovskites, spinels, chalcogenides, and others to accelerate the discovery of novel materials with extreme physical properties.

## 4 Methodology

### 4.1 DFT Training Dataset Generation

**FIGURE 9:**
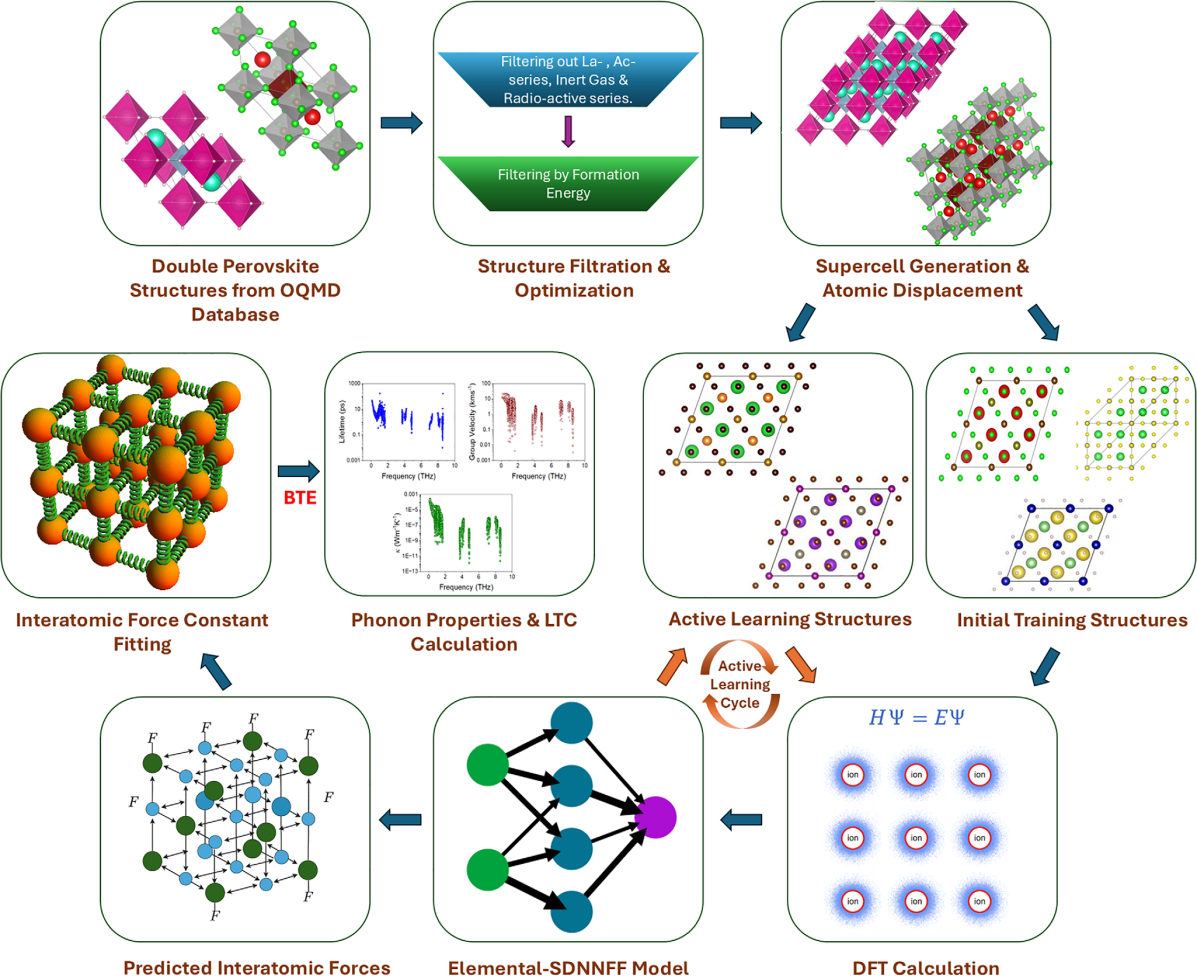
Workflow of developing deep learning Elemental‐SDNNFF model for discovering ultralow thermal conductivity double perovskites. The blue lines represent the backbone of the workflow, whereas the orange lines represent the active learning loop. The IFCs are fitted after getting the forces from the finally frozen Elemental‐SDNNFF model, followed by the calculation of lattice thermal conductivity and other related phonon properties.

The entire workflow of developing deep learning Elemental‐SDNNFF model for discovering double perovskites with ultralow thermal conductivity is presented in Figure 9. In this study, cubic double perovskites structures with different compositions were acquired from Open Quantum Materials Database (OQMD) [57]. Initial dataset filtration is done by screening out those with chemical formula match double perovskites generic configuration (ABC_2_D_6_) and excluding the structures containing lanthanide or actinide elements. These structures are then re‐optimized using DFT with our own high accuracy parameters. For instance, the convergence criteria for energy and atomic force are set as 10^−8^ eV and 10^−4^ eV/Å, respectively. For electrons the product of k‐points along a specific crystallographic direction of the primitive cell and corresponding lattice constant in Angstrom is set as 80. After structure re‐optimization, further filtering is done by considering formation energy. Negative formation energy value increases the possibility of the stability of a structure. A total of 9,709 successfully re‐optimized structures is collected after the filtration. Afterwards, the primitive cells are replicated by 2 × 2 × 2 for all structures to create the supercells, and then the atoms in the supercells are randomly displaced in different directions by a constant displacement of 0.03 Å using PHONOPY [95] package. 100 such displaced supercells are generated for each structure, resulting in a total of 970 900 displaced supercells. High‐precision self‐consistent field DFT calculations are performed with an energy convergence threshold of 10^−6^ eV.

### 4.2 Deep Learning Elemental‐SDNNFF Model Training

In this study, Elemental‐SDNNFF deep learning model is trained on the displaced atomic position and corresponding forces. [32] In Elemental‐SDNNFF model, atomic configurations are rotated by nearest neighbor rule for data augmentation. [33] 16 396 atomic configurations for different double perovskites are randomly selected from the original pool of 970 900 displaced supercells and the corresponding forces are calculated by DFT, and then these 16 396 configurations with 1 311 680 atomic force entries (each configuration contains 80 atoms) serve as initial training dataset. Six Elemental‐SDNNFF models are trained in parallel with the same training dataset, and after training, these six models are then used for evaluating all other displaced atomic configurations which were not used in the initial training, during which the maximum uncertainty for forces is calculated by [96]

(4) $$\varepsilon_{i}=\max_{i}\;\sqrt{{\langle \left\|f_{i}^{m}-\bar{f_{i}}\right\|\rangle }^{2}}$$

where, ε_*i*_ is the maximum uncertainty, $f_{i}^{m}$ is the predicted force by the trained model, and $\bar{f_{i}}$ is the average force across all models in the committee. This iterative model training process was called the active learning process and the whole training process was shown in Figure 9. The model uncertainty was quantified for each displaced supercell, and those with the highest uncertainty were prioritized for additional DFT calculations. Approximately 670 such supercells were selected, and the resulting data were added to the training set. The models were then fine‐tuned iteratively in this active learning cycle, summarized in Figure 9. [70] When the RMSE of the predicted forces compared to DFT forces converges to a nearly constant value, the model is finally frozen and the final published model is used for force evaluation for all remaining 950 467 atomic configurations, which are not used in any previous rounds of the training. The final reported predicted phonon properties are calculated by randomly selecting 100 configurations for each material among these remaining atomic configurations and performing IFC fitting and then obtaining LTC as described above.

### 4.3 Solving BTE for Phonon Transport Properties

Comprehensive phonon transport properties were obtained by solving phonon BTE with IFCs as input and the calculation was done by ShengBTE package. For 3 ph only, NGRID size in the range of 14 × 14 × 14 to 20 × 20 × 20 were used for all double perovskites, depending on the size of the lattice parameters. The chosen NGRID size guarantees the total number of scattering channels in the material exceeding 10^8^ and thus large enough to achieve converged LTC results. For 3ph + 4 ph BTE calculation, the NGRID size has to be reduced to 6 × 6 × 6, due to the large number of phonon scattering channels in 4 ph scattering and thus leading to significantly longer computation time. For the lowest LTC material Cs_2_HgPtCl_6_, we also tested the NGRID size of 6 × 6 × 6 for 3 ph only and got almost similar LTC results as 3 ph + 4 ph. That means for this specific material, the thermal conductivity is not sensitive to the NGRID size for 3 ph scattering, perhaps it is because the material has extremely low thermal conductivity. However, we must emphasize that this conclusion might not be applicable to other materials.

### 4.4 DeePMD Interatomic Potential Training

Initially, ab initio molecular dynamics (AIMD) simulations with electron k‐points sampled at Г‐point only were performed for the Cs_2_HgPtCl_6_ structure at 6 different temperatures ranging from 300 to 800 K in 100 K increments. Afterwards, 205 different atomic configurations are randomly sampled from each AIMD simulation, resulting in a total of 1230 configurations ready to use for further high precision DFT calculations. These sampled structures were then re‐calculated by high precision self‐consistent field DFT calculations with dense k‐points mesh to obtain the forces, energies, and virials. The process followed the same general methodology outlined in the “DFT Training Dataset Generation” section. All these DFT calculated data are served as the input for the training set. Here, “dpa‐2” [97] deep potential (DP) model from DeePMD‐kit package [98, 99] is employed for the training. The DeepMD model is trained for 10^6^ epochs, achieving a RMSE of approximately 4 to 5 meV/Å for the forces and 0.04 meV/atom for energies. Finally, DeePMD‐kit has been used to freeze the interatomic potential, which is subsequently used in MD simulation performed with LAMMPS [100] package to capture the dynamic behavior of Cs_2_HgPtCl_6_.

### 4.5 Extract Phonon Lifetime from MD Trajectory

For this part of the study, MD simulation is performed with DeepMD‐equipped LAMMPS package conducted using NVT ensembles for 5000 steps to equilibrate the system at each specific temperature (ranging from 100 to 700 K in 100 K increments), followed by 20 000 steps using NVE ensemble to generate the trajectory file. The atomic trajectories generated from the NVE simulation are used as input for DynaPhoPy. DynaPhoPy analyzes this trajectory file and renormalizes the force constants, which is eventually used to calculate the phonon linewidth by accounting anharmonic correction. [38] Finally, phonon mode level lifetime is calculated by [94],  

(5) $$\tau =\frac{1}{2\Gamma }\;$$

where Γ is the phonon linewidth extracted from MD simulation. The lattice thermal conductivity is then re‐calculated by Equation (1) with phonon frequency and group velocity calculated by the renormalized force constants and relaxation time obtained from Equation (5). The temperature dependent thermal conductivity result obtained by this method is included in Figure 4 for validation of DFT+BTE approach and denoted as “DynaPhoPy+DeepMD”.

## Author Contributions

**M.H**. conveyed the idea and designed and supervised the study. **M.Z.A**. and **A.R**. developed the Elemental‐SDNNFF model and performed model evaluation and testing. **M.Z.A**. performed DeePMD training and testing and extracted phonon lifetime from MD simulations using the DynaPhoPy package. **R.R**. conducted significant DFT calculations for dynamic stability validation. **M.Z.A**. prepared the draft of the manuscript. **M.H**. and **R.R**. revised the manuscript. All authors contributed to discussions and interpretation of results in the manuscript.

## Conflicts of Interest

The authors declare no conflict of interest.

## Supporting information




**Supporting File**: advs73904‐sup‐0001‐SuppMat.docx.

## Data Availability

The data that support the findings of this study are available in the supplementary material of this article.
