# Supplementary material for: Machine Learning Discovery of Record‐Low Lattice Thermal Conductivity in Double Perovskites
Source: Adv Sci (Weinh). 2026 Feb 17;13(18):e15766. doi: 10.1002/advs.202515766 (PMC13042595; doi:10.1002/advs.202515766)
Supplement: Supplementary file 1 — Supporting File: advs73904‐sup‐0001‐SuppMat.docx. [file ADVS-13-e15766-s001.docx]

*Supplementary Information for*

**Machine Learning Discovery of Record-Low Lattice Thermal Conductivity in Double Perovskites**

Md Zaibul Anam,^1^ Alejandro Rodriguez,^1^ Riccardo Rurali,^2^ and Ming Hu^1,^^[[1]](#footnote-1)^*

^1^Department of Mechanical Engineering, University of South Carolina, Columbia, SC 29208, USA

^2^Institut de Ciència de Materials de Barcelona, ICMAB–CSIC, Campus UAB, 08193 Bellaterra, Spain

**Table S1.** List of double perovskite structures screened in this study with full LTC information by considering both 3-phonon and 4-phonon scattering. Note that all materials are cubic structures with space group no. 225, so the thermal transport is isotropic in all 3 directions and therefore only single value for LTC is reported here. All LTC values are in units of Wm^-1^K^-1^.

| Structure ID | Formula | *κ_p_* (3ph) | *κ_c_* (3ph) | *κ_Total_* (3ph) | *κ_p_* (3ph+4ph) | *κ_c_* (3ph+4ph) | *κ_Total_* (3ph+4ph) |
| --- | --- | --- | --- | --- | --- | --- | --- |
| 1370196 | Cs_2_KRhCl_6_ | 0.079 | 0.297 | 0.376 | 0.006 | 0.908 | 0.913 |
| 1549329 | Cs_2_HgPtCl_6_ | 0.267 | 0.078 | 0.345 | **0.018** | **0.053** | **0.071** |
| 1575131 | Cs_2_InRhBr_6_ | 0.215 | 0.159 | 0.374 | 0.045 | 0.355 | 0.401 |
| 1575138 | Cs_2_AgOsBr_6_ | 0.088 | 0.143 | 0.231 | 0.014 | 0.049 | 0.063 |
| 1720380 | Cs_2_KIrCl_6_ | 0.198 | 0.183 | 0.381 | 0.007 | 1.343 | 1.350 |
| 1368340 | Cs_2_NaCdF_6_ | 0.386 | 0.434 | 0.820 | 0.015 | 0.848 | 0.863 |
| 1431102 | Cs_2_NaHgF_6_ | 0.627 | 0.201 | 0.828 | 0.028 | 0.176 | 0.204 |
| 1288224 | Cs_2_RbAlF_6_ | 0.723 | 0.244 | 0.968 | 0.027 | 1.093 | 1.120 |
| 1209420 | Cs_2_RbAlF_6_ | 0.740 | 0.259 | 0.999 | 0.046 | 1.362 | 1.408 |
| 1430862 | Cs_2_NaHgF_6_ | 0.635 | 0.460 | 1.094 | 0.033 | 0.894 | 0.927 |
| 1430830 | Cs_2_AgRhF_6_ | 1.059 | 0.165 | 1.225 | 0.084 | 0.235 | 0.319 |
| 1429141 | Rb_2_OsPbO_6_ | 0.424 | 0.938 | 1.362 | 0.010 | 1.259 | 1.269 |
| 1369030 | KRb_2_AlH_6_ | 0.738 | 0.631 | 1.369 | 0.232 | 1.664 | 1.896 |
| 1428989 | K_2_TlH_6_Rh | 0.908 | 0.541 | 1.449 | 0.078 | 0.853 | 0.931 |
| 1288223 | Cs_2_KAlF_6_ | 1.297 | 0.171 | 1.468 | 0.114 | 1.045 | 1.159 |
| 1209421 | Cs_2_KAlF_6_ | 1.318 | 0.167 | 1.485 | 0.187 | 0.735 | 0.921 |
| 1365379 | Cs_2_RbCoH_6_ | 0.991 | 0.513 | 1.504 | 0.150 | 1.038 | 1.188 |
| 1368245 | Cs_2_RbAlH_6_ | 1.460 | 0.077 | 1.537 | 0.059 | 0.151 | 0.210 |
| 1431250 | Sr_2_H_6_IrPt | 0.621 | 0.206 | 0.827 | 0.117 | 0.301 | 0.419 |
| 1431444 | Ba_2_H_6_RhAu | 0.741 | 0.088 | 0.829 | 0.065 | 0.161 | 0.225 |
| 1432305 | Rb_2_LiAlCl_6_ | 0.248 | 0.664 | 0.913 | 0.037 | 0.800 | 0.837 |
| 1428799 | Cs_2_NaCoCl_6_ | 0.719 | 0.194 | 0.913 | 0.041 | 0.488 | 0.529 |
| 1720995 | Rb_2_LiAlCl_6_ | 0.253 | 0.663 | 0.916 | 0.018 | 0.814 | 0.832 |
| 1432599 | Cs_2_NaRhCl_6_ | 0.784 | 0.153 | 0.936 | 0.029 | 0.544 | 0.573 |
| 1261041 | Ba_2_HfAlS_6_ | 0.987 | 0.392 | 1.378 | 0.118 | 0.455 | 0.574 |
| 1543168 | Cs_2_KRhCl_6_ | 0.078 | 0.299 | 0.377 | 0.003 | 0.876 | 0.879 |
| 1549349 | Cs_2_KRuCl_6_ | 0.120 | 0.261 | 0.381 | 0.002 | 0.795 | 0.797 |
| 1546761 | Cs_2_TlRhBr_6_ | 0.081 | 0.304 | 0.385 | 0.002 | 0.618 | 0.619 |
| 1575778 | Cs_2_KIrCl_6_ | 0.113 | 0.289 | 0.402 | 0.003 | 0.812 | 0.815 |
| 1432641 | Cs_2_KAlCl_6_ | 0.200 | 0.244 | 0.445 | 0.014 | 0.907 | 0.921 |
| 1543122 | Rb_2_NaCoCl_6_ | 0.104 | 0.384 | 0.487 | 0.009 | 0.837 | 0.846 |
| 1432196 | Rb_2_NaCoCl_6_ | 0.100 | 0.400 | 0.501 | 0.009 | 1.052 | 1.061 |
| 1543163 | Cs_2_NaHfCl_6_ | 0.362 | 0.196 | 0.558 | 0.009 | 0.635 | 0.644 |
| 1721624 | Cs_2_InIrCl_6_ | 0.567 | 0.064 | 0.631 | 0.016 | 0.084 | 0.100 |
| 1432636 | Cs_2_KCoCl_6_ | 0.542 | 0.162 | 0.704 | 0.005 | 0.822 | 0.827 |
| 1575765 | Cs_2_InRhCl_6_ | 0.750 | 0.066 | 0.816 | 0.044 | 0.111 | 0.156 |
| 1575133 | Cs_2_AlAgBr_6_ | 0.146 | 0.299 | 0.445 | 0.030 | 0.416 | 0.446 |
| 1541451 | Cs_2_AgPbCl_6_ | 0.315 | 0.258 | 0.573 | 0.031 | 0.292 | 0.324 |
| 12030 | Cs_2_LiYCl_6_ | 0.356 | 0.293 | 0.649 | 0.037 | 0.250 | 0.286 |
| 1546965 | Rb_2_LiIrCl_6_ | 0.100 | 0.597 | 0.697 | 0.006 | 0.927 | 0.933 |
| 1432574 | Cs_2_AgRhCl_6_ | 0.455 | 0.266 | 0.721 | 0.066 | 0.489 | 0.554 |
| 1432319 | Rb_2_LiCoCl_6_ | 0.083 | 0.694 | 0.776 | 0.004 | 0.650 | 0.654 |
| 1428955 | Sr_2_H_6_IrAu | 0.646 | 0.416 | 1.063 | 0.065 | 1.573 | 1.638 |
| 1431315 | K_2_CdH_6_Rh | 0.740 | 0.468 | 1.208 | 0.074 | 1.124 | 1.198 |
| 1431247 | Sr_2_H_6_RhAu | 1.167 | 0.121 | 1.288 | 0.030 | 0.191 | 0.221 |
| 1431218 | Sr_2_H_6_IrPd | 1.364 | 0.254 | 1.617 | 0.289 | 0.753 | 1.043 |
| 1344196 | Cs_2_LiBiCl_6_ | 0.176 | 0.492 | 0.668 | 0.020 | 0.507 | 0.527 |


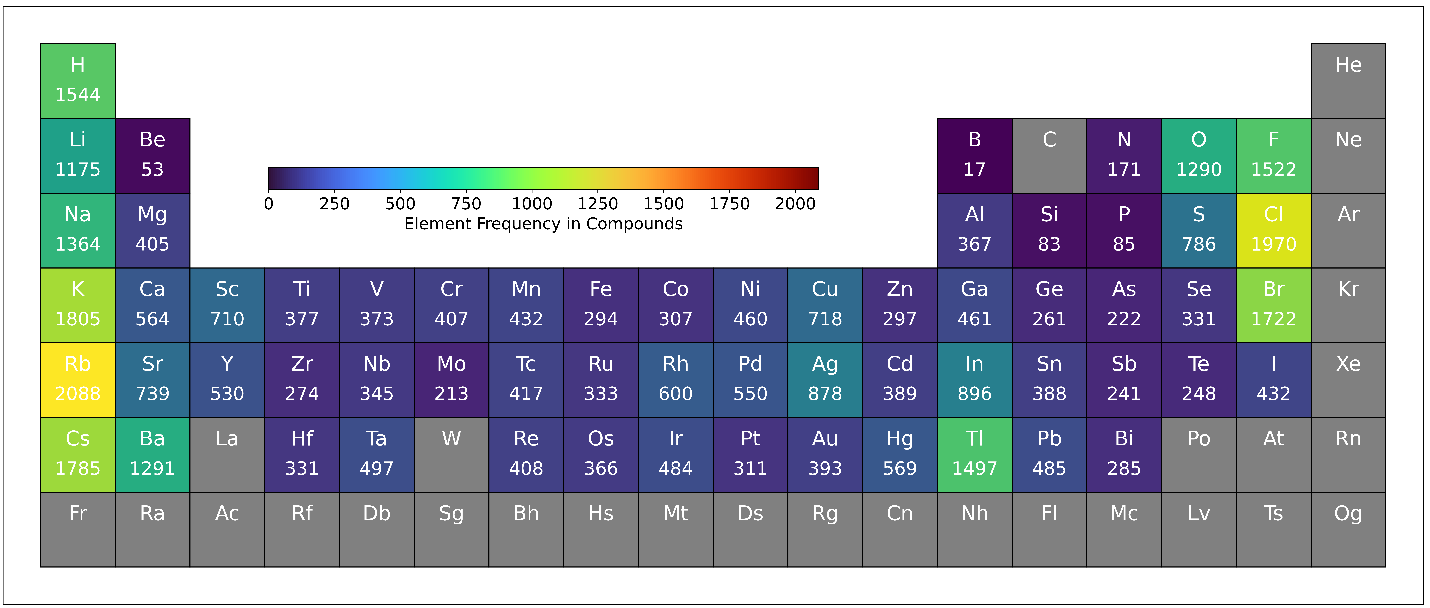


**Figure S1:** Element occurrence of the screening pool of 9,709 double perovskite structures across the periodic table.


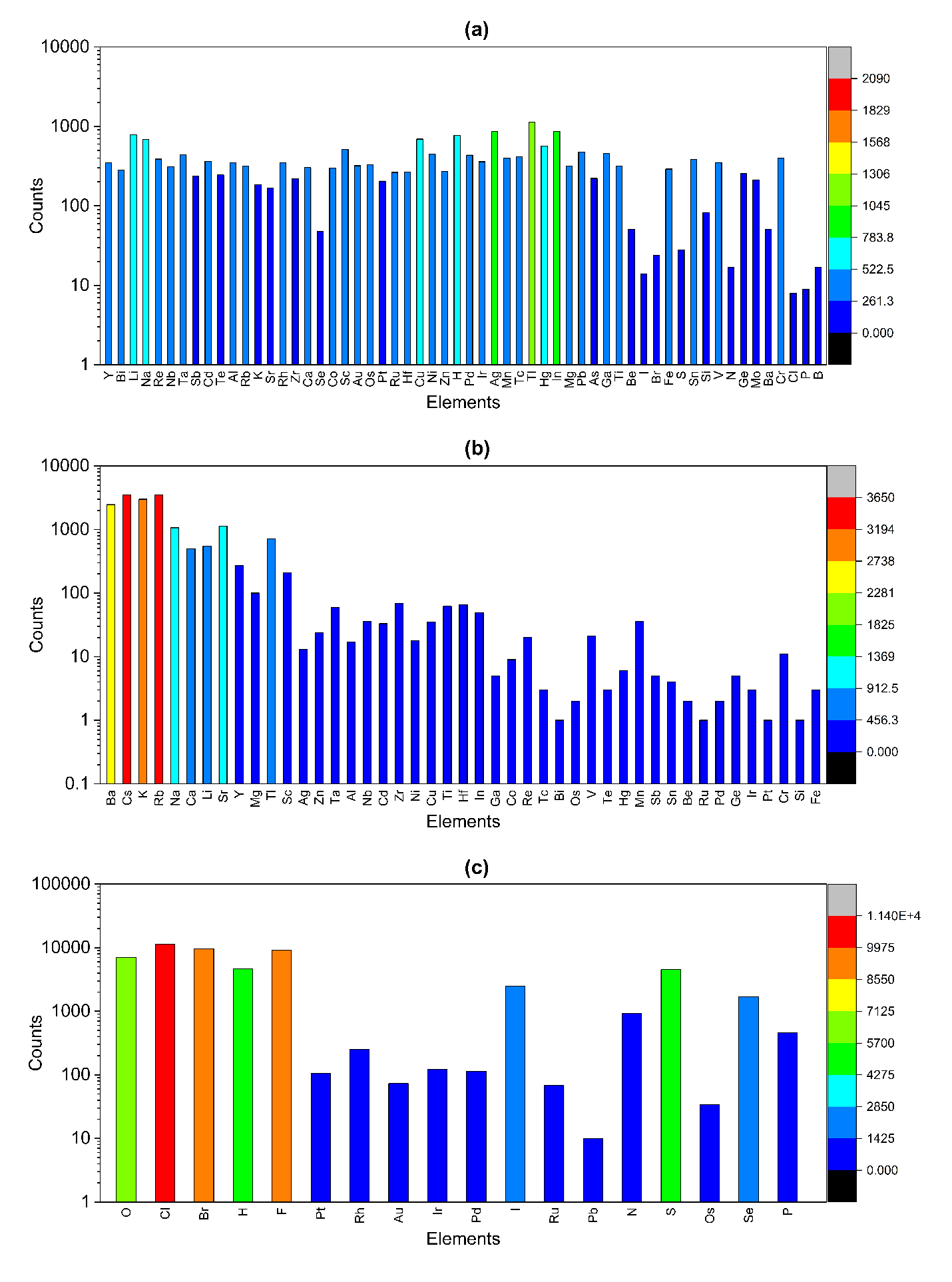


**Figure S2:** Element distribution statistics of double perovskite compounds following the generic formula ABC_2_D_6_. The frequency of elements occupying the (a) A and B site, (b) C site, and (d) D site are shown, and the number occurrence for each element is highlighted with the color bars.


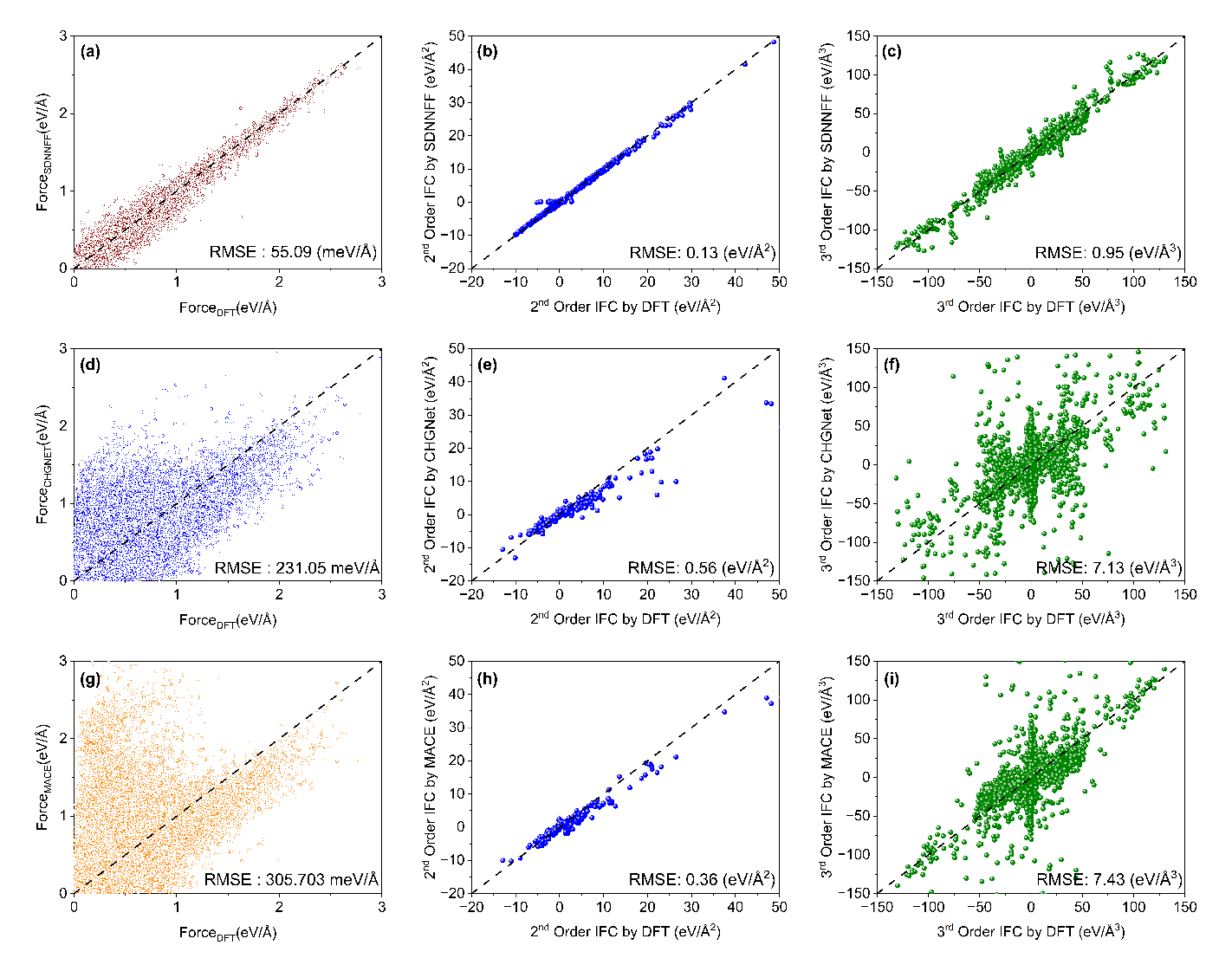


**Figure S3:** Comparison of atomic forces (a,d,g), 2^nd^ order (b,e,h), and 3^rd^ order (c,f,i) interatomic force constants for Elemental-SDNNFF, CHGNet, and MACE model. The dashed line represents the perfect correlation between DFT and predicted data.


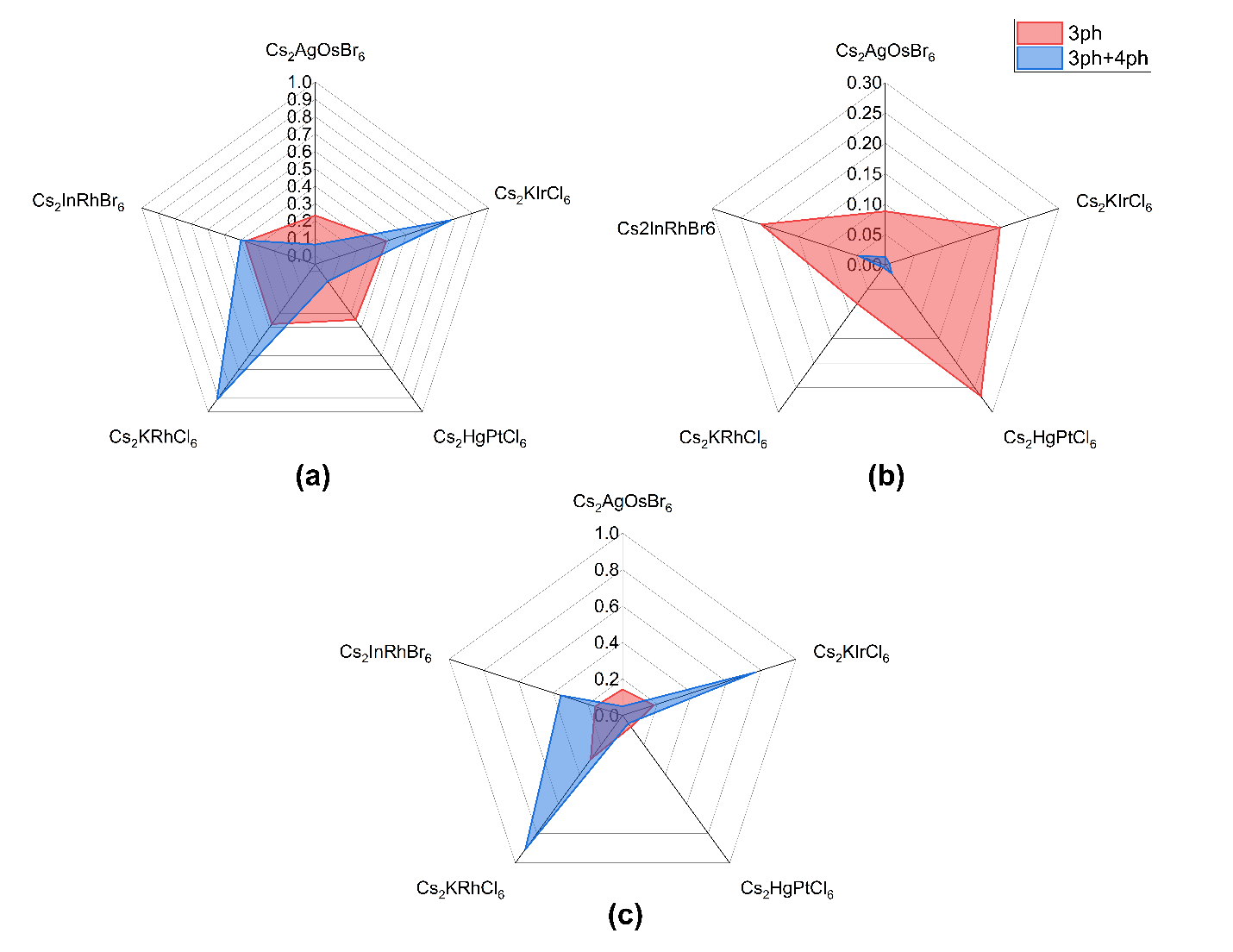


**Figure S4:** Comparison of the effect of three-phonon (3ph) and combined three- plus four-phonon (3ph+4ph) on (a) $\kappa_{total}$, (b) $\kappa_{p}$, and (c) $\kappa_{c}$ for 5 selected structures: Cs_2_AgOsBr_6_, Cs_2_InRhBr_6_, Cs_2_KRhCl_6_, Cs_2_HgPtCl_6_, and Cs_2_KIrCl_6_. The red and blue shaded region represents values obtained using 3ph and 3ph+4ph process, respectively.

**
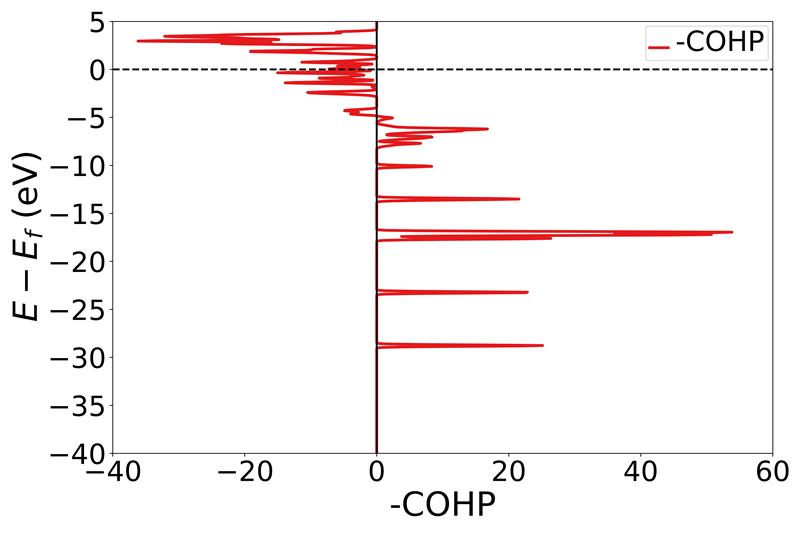
**

**Figure S5.** The crystal orbital Hamilton population (COHP) plots for Cs_2_HgPtCl_6_.

1. *Author to whom all correspondence should be addressed. E-Mail: [hu@sc.edu](mailto:hu@sc.edu) [↑](#footnote-ref-1)
